# Predictors of Moral Distress Among Nurses: A Cross-Sectional Study

**DOI:** 10.3390/ijerph23060761

**Published:** 2026-06-05

**Authors:** Vladimír Siska, Andrea Sollárová, Zuzana Slezáková, Lukáš Kober, Peter Minárik, Tomáš Forgon

**Affiliations:** 1 Department of Nursing, Faculty of Nursing and Health Professional Studies, Slovak Medical University in Bratislava, 833 03 Bratislava, Slovakia; zuzana.slezakova@szu.sk; 2 Department of Nursing, Faculty of Social Sciences and Health Care, Constantine the Philosopher University in Nitra, 949 01 Nitra, Slovakia; asollarova@ukf.sk (A.S.); pminarik@ukf.sk (P.M.); tforgon@ukf.sk (T.F.); 3 Department of Nursing, Faculty of Health Sciences, Catholic University in Ružomberok, 034 01 Ruzomberok, Slovakia; lukas.kober@ku.sk

**Keywords:** moral distress, nursing, ethics

## Abstract

**Highlights:**

**Public health relevance—How does this work relate to a public health issue?**

- This study addresses moral distress among nurses, which represents a significant public health concern, as nurses play a key role in the provision of healthcare. Moral distress can lead to psychological exhaustion, negatively affecting the quality of patient care and contributing to a higher risk of burnout and nurses leaving the profession.
- The issue of moral distress has not been sufficiently explored in the context of the Slovak Republic.

**Public health significance—Why is this work of significance to public health?**

- Prevention of burnout in nurses and potential attrition from the profession.
- Empirical evidence on factors contributing to moral distress. Identifying these predictors enables the development of targeted interventions and programs that can strengthen nurses’ mental health and professional satisfaction. This not only improves their well-being but also enhances the quality of patient care, which is essential for public health.

**Public health implications—What are the key implications or messages for practitioners, policy makers and/or researchers in public health?**

- Support for legislation and policies: Policymakers should consider implementing programs focused on the mental well-being of healthcare professionals as part of broader healthcare improvement strategies.
- Importance of research: This study encourages further research on moral distress and its impact on healthcare, which is essential for developing effective public health strategies.

**Abstract:**

Investigating the predictors of moral distress is particularly important for protecting nurses’ mental health and professional satisfaction, thereby preventing burnout and attrition from the profession. The primary aim of this study was to evaluate the predictors of moral distress among nurses. A cross-sectional study design was used. The sample consisted of 412 nurses from 11 hospitals across Slovakia. The mean age of the respondents was 40.48 years (SD = 10.92). Moral distress was assessed using the Modified Moral Distress Scale. Linear regression analysis was used to evaluate predictors of moral distress. Personal accomplishment, the maladaptive coping strategy of self-distraction, continuous shift operation, and emotional burnout emerged as significant predictors of the frequency of moral distress among nurses (AdjR^2^ = 15.5%; R^2^ = 0.155). Regarding the intensity of moral distress, significant predictors included personal accomplishment, the maladaptive strategy of self-distraction and the adaptive strategy of religiosity and spirituality (AdjR^2^ = 14.0%; R^2^ = 0.140), which appear to function as adaptive coping mechanisms for dealing with the intensity of moral distress. Systematic investigation of predictors of moral distress among nurses may contribute to the development of interventions and programs that support nurses, thereby improving not only their job satisfaction but also the quality of patient care.

## 1. Introduction

The phenomenon of moral distress has gained increasing attention in nursing practice, education, and research. Nearly 47% of nurses working in acute care hospital units frequently encounter situations that trigger moral distress [1]. The literature presents various definitions and perspectives on moral distress [2,3]. Moral distress is currently understood as a complex, multilevel, and dynamic phenomenon that arises in situations where healthcare professionals recognize the ethically appropriate course of action but are unable to act in accordance with this judgment due to various constraints [4]. This foundational concept has been further expanded to include additional dimensions reflecting its complexity and contextual nature [5]. From a conceptual perspective, moral distress can be interpreted through the interaction of three key domains: (1) organizational, (2) individual, and (3) ethical. The organizational domain includes structural and systemic factors influencing care delivery [6], while the individual domain encompasses personal characteristics such as personality traits [7]. An important component of this conceptual framework is its dynamic and cumulative nature. Repeated exposure to morally distressing situations leads to the accumulation of so-called moral residue and may result in the “crescendo effect,” characterized by a progressive intensification of psychological burden [8].

### Theoretical Model of the Study

The model assumes that moral distress arises as a result of the interaction of multiple factors. In this study, it is operationalized through the dimensions of frequency and intensity, which corresponds to the approach used in instruments such as the MDS [9]. Organizational factors (e.g., working hours, workplace) represent a source of ethically challenging situations and play a key role in the development and mitigation of moral distress [10,11,12]. Individual characteristics (age, years of practice) influence the perception and accumulation of moral distress, with the accumulation of moral distress playing a significant role [13]. Personality factors (Big Five) determine emotional reactivity and sensitivity to ethical conflicts; for example, a negative personality profile was associated with a significantly higher prevalence of moderate to severe moral distress [14]. Coping strategies act as a moderating factor—maladaptive strategies (e.g., self-distraction) may increase distress, whereas adaptive strategies (e.g., spirituality) may reduce it [15]. A key mechanism in the model is burnout, which is closely linked to moral distress, particularly in the dimension of emotional exhaustion [16,17]. The model therefore assumes:

Organizational factors + individual and personality characteristics → coping and burnout → moral distress (frequency, intensity).

This approach conceptualizes moral distress as a multifactorial and dynamic phenomenon shaped by both individual and systemic factors [18].

Nursing care is inherently associated with ethical issues [19]. Currently, nurses experience moral distress more frequently than in the past due to clinical errors and staff shortages, as well as conflicts between individual values and job demands, administrative policies, managerial decisions, and workplace regulations [20,21,22]. If left unaddressed, moral distress threatens care at both the individual level (as a cause of burnout, staff turnover, demoralization, and desensitization) and the system level (reduced quality of care), thereby compromising the integrity of healthcare professionals, healthcare systems, and patient safety [23,24,25].

When nurses are exposed to prolonged moral distress, they may begin to avoid certain aspects of patient care, distancing themselves from patients, becoming emotionally unavailable, or avoiding entering patients’ rooms [26,27]. The sources of moral distress may vary and can stem from clinical conditions, the work environment, and both external and internal factors [28].

External factors primarily include inadequate staffing, lack of time, insufficient administrative support, and institutional policies and priorities that may conflict with patient care [29]. Internal factors relate to, for instance, personal aspects that influence the perceived ability of providers to deliver optimal care. These internal factors include fear of job loss, anxiety, feelings of helplessness, a lack of assertiveness, insufficient understanding of the situation, and low self-esteem [30]. Risk factors for moral distress include not only nursing staff shortages but also insufficient experience and education, poor teamwork between physicians and nurses, high workload, and low quality of care [31]. Previous research suggests that moral distress among nurses may be more strongly associated with older age and longer work experience [13], and less associated with gender or religion [32].

The main aim of this study was to evaluate selected demographic and personality characteristics as predictors of moral distress among nurses working in various clinical settings within our sociocultural context.

The importance of investigating moral distress in the Slovak sociocultural context is based on the assumption that although moral distress in nurses is a globally recognized phenomenon, its determinants and consequences are significantly modified by the cultural, organizational and systemic environment of healthcare. Most of the research to date comes mainly from non-European countries such as North America, the Middle East, Asia. From European countries, these are Western European and Northern European countries [12]. These are countries with different working conditions, professional autonomy of nurses, staffing and models of interdisciplinary cooperation compared to the Central European countries, within which we present new findings. In the Slovak healthcare system, moral distress can be specifically influenced by factors such as long-term staff shortages, high workload, lower participation of nurses in decision-making processes, hierarchical model of work relationships, limited personnel and material resources, as well as culturally conditioned expectations regarding the professional role of nurses. These characteristics can change not only the frequency of morally stressful situations, but also the way they are psychologically processed and their relationship to burnout or workload.

The contribution of our research also lies in the fact that moral distress is not examined in isolation, but in relation to personality characteristics, working conditions, burnout syndrome and subjectively perceived workload of nurses. This complex model allows for a better understanding of which individual and organizational factors can increase the vulnerability of nurses to moral distress under the conditions of the Slovak healthcare system. At the same time, it can contribute to the development of more targeted prevention and intervention strategies aimed at supporting the mental health of nurses and the sustainability of the nursing profession.

## 2. Materials and Methods

### 2.1. Study Design and Setting

The study was approved by the ethics committees of the participating healthcare institutions. Data collection commenced in July 2025 after approval had been obtained from the first participating institution. The research proposal was not approved by two inpatient healthcare institutions; therefore, data collection was not conducted at these sites. In all eleven participating healthcare institutions, data collection in every institution began only after ethics committee approval and informed consent from the respondents had been obtained. Data collection finished by the end of October 2025.

The study utilized a cross-sectional design. The inclusion criteria were the type of care unit (surgical, internal medicine, anesthesiology, or intensive care), provision of care to adult patients (aged 19 years and older), and provision of informed consent. The exclusion criteria included care of pediatric patients, managerial positions, outpatient care, and lack of informed consent.

The power analysis using G*Power software (ver. 3.1.9.7) suggested a minimum sample size of *n* = 249 participants to reliably detect a medium effect in a multiple regression model with 27 predictors at α = 0.05 and power = 0.95. Given the number of predictors included in the model, a medium effect size was assumed, reflecting the expectation that individual predictors would contribute modestly to the explained variance. The final sample consisted of 412 nurses, exceeding this threshold and thus resulting in increased statistical power and improved stability of the regression estimates. The relatively high number of predictors suggests that a larger sample may further improve model stability; it may also contribute to more precise effect estimates.

### 2.2. Research Measurements

Data on the occurrence of moral distress were collected using the Modified Moral Distress Scale (MDS-11) [9]. Permission to use the scale and translate it into Slovak was obtained from the authors. A double back-translation was carried out by two independent English-speaking experts. Subsequently, the translated version of the questionnaire was content-validated by four experts in the fields of nursing and psychology. After reaching consensus, the Slovak version of the questionnaire was finalized and subjected to pilot testing. The scale consists of 11 items examining moral distress experienced by nurses in clinical practice and is appropriate for use across various clinical settings. The frequency of moral distress is assessed on a five-point Likert scale ranging from “1—never” to “5—always,” and the intensity of moral distress is measured on a five-point Likert scale ranging from “1—none” to “5—severe.” Lower scores indicate mild moral distress experienced by nurses in clinical practice, while higher scores indicate the perception of extreme distress. Exploratory factor analysis (principal component extraction) supported a one-factor solution explaining 31.5% of the variance in the frequency domain and 37.8% of the variance in the intensity domain. All items loaded positively on the single component, with factor loadings ranging from 0.41 to 0.64 and 0.48 to 0.69, respectively, indicating a coherent unidimensional structure of both domains of moral distress scale. The reliability of the original version ranges from α = 0.76 to 0.82 [9]. Cronbach’s alpha coefficients in our data were 0.78 in the frequency domain and 0.83 in the intensity domain, indicating high reliability.

The International Personality Item Pool (Mini-IPIP) questionnaire [33], Slovak version [34], was used to assess personality traits according to the Big Five theory (extraversion, emotional stability, openness to experience, agreeableness, and conscientiousness). The questionnaire consists of 20 items, i.e., four items per factor. Items are rated on a Likert scale from “1—does not describe me at all” to “5—describes me completely.” The reliability of all five factors for the Slovak version ranges from α = 0.73 to 0.90 [35]. Cronbach’s alpha coefficients in our data were from α = 0.64 to 0.81.

The Maslach Burnout Inventory—Human Services Survey (MBI-HSS) [36], Slovak version [37], was used to assess subjective levels of burnout among helping professionals. It consists of 22 items describing various positive and negative feelings that may be experienced when working with people, with responses on a 7-point scale ranging from “0—never” to “6—every day,” depending on how frequently these feelings are experienced. The items load onto three factors: emotional exhaustion, depersonalization, and reduced personal accomplishment. Higher scores indicate higher levels of burnout. Previous research has reported Cronbach’s alpha coefficients of approximately α = 0.85–0.90 for emotional exhaustion, α = 0.65–0.80 for depersonalization, and α = 0.70–0.85 for personal Accomplishment [24]. Cronbach’s alpha coefficients in our data were α = 0.75 for emotional exhaustion, α = 0.71 for depersonalization, and α = 0.74 for personal accomplishment.

Coping strategies were assessed using the Brief COPE questionnaire [38], Slovak version [39]. The questionnaire contains 28 items divided into 14 subscales representing problem-focused, emotion-focused, and avoidance coping strategies. Items are rated on a four-point scale: “0—yes,” “1—rather yes,” “2—rather no,” “3—no,” with respondents indicating how they behaved in stressful situations over the past six months. The reliability of the original version for individual subscales ranged from approximately α = 0.50 to 0.90 [38] and in our data, it ranged from α = 0.32 to 0.90. Internal consistency of the Brief COPE subscales ranged from low to good, which is consistent with previous research [38], given that each subscale consists of only two items. However, lower reliability coefficients (α < 0.50) were observed for several subscales, particularly Active coping, Denial, Behavioral disengagement, Venting, and Self-blame. Therefore, findings related to these subscales should be interpreted with caution due to the increased risk of measurement error and reduced stability of regression estimates.

### 2.3. Data Analysis

Data analysis was performed using IBM SPSS Statistics 21.0. Both descriptive and inferential statistics were applied. Linear regression analysis was used to evaluate predictors (personality factors, burnout levels, and coping strategies) of moral distress. Stepwise regression was employed as an exploratory approach to identify the most significant and parsimonious set of predictors of moral distress from multiple potentially interrelated variables, particularly in the absence of strong theoretical guidance on their relative importance.

All available demographic and professional variables (age, gender, education, years of practice, and unit type) were entered into the initial stepwise regression model. However, as stepwise procedures retain variables based on statistical criteria, this approach does not guarantee full control of confounding. This is now acknowledged as a limitation in the manuscript.

Residual diagnostics indicated that the residuals were centered around zero (M = 0.00), suggesting no systematic bias. Standardized residuals ranged from −2.46 to 4.02, indicating the presence of a small number of potential outliers. However, given the large sample size, their impact is unlikely to substantially affect the results. Multicollinearity was not a concern (VIFs ≈ 1.00–1.12). Visual inspection of the scatterplot of standardized residuals against standardized predicted values indicated no evidence of heteroscedasticity.

## 3. Results

A total of 1100 questionnaires were distributed, with 462 returned (response rate 37.45%). Of the returned questionnaires, 50 were excluded due to incomplete responses or because they were completed by respondents in managerial positions, which constituted an exclusion criterion. The sample consisted of 412 nurses from 11 hospitals across Slovakia. The mean age of the respondents was 40.48 years (SD = 10.92). A more detailed description of the sample in terms of demographic and professional variables is presented in Table 1.

The results present findings related to the predictors of moral distress among nurses. The results examine the associations between the frequency and intensity of moral distress and demographic characteristics, personality traits, burnout level, and coping strategies using multivariable regression models. The initial regression models included demographic and professional variables (age, gender, education, years of practice, and unit type), personality traits (five variables), coping strategies (14 variables), and burnout dimensions (three variables). All variables were entered into the models, and a stepwise procedure was used to identify predictors retained in the final model. The findings indicate four models predicting the frequency of moral distress among nurses.

Table 2 presents the overall evaluation of four models predicting the frequency of moral distress among nurses. The first model identifies personal accomplishment as the strongest predictor among the examined demographic and personality factors (Table 3). This model explains 12.6% of the variance in the dependent variable (R^2^ = 0.126).

The second model includes, in addition to personal accomplishment, the maladaptive coping strategy of self-distraction. This model explains 14.5% of the variability in the dependent variable (R^2^ = 0.145). The third model incorporates personal accomplishment, self-distraction, and shift work, explaining 15.5% of the variance in the dependent variable (R^2^ = 0.155).

A fourth statistically significant model is further enriched by the variable emotional burnout and explains 16.4% of the variability in the frequency of moral distress among nurses. Other examined demographic and personality characteristics did not contribute to explaining the frequency of moral distress among nurses.

The frequency of moral distress experienced by nurses is most strongly predicted by personal accomplishment, self-distraction, shift work, and emotional burnout. These represent a combination of selected dimensions of burnout, coping strategies, and work regime (shift work). Their higher presence in the demographic and personality profile of respondents/nurses predicted a higher frequency of moral distress. However, the total explained variance remains moderately low, supporting the interpretation that moral distress is a multifactorial phenomenon with a significant contribution of other (primarily organizational) determinants.

In the second part of the results, the relationship between the intensity of moral distress and demographic characteristics, personality traits, burnout levels, and coping strategies is examined. The findings indicate four models predicting the frequency of moral distress among nurses.

Table 4 presents the overall evaluation of three models predicting the intensity of moral distress among nurses. The first model identifies personal accomplishment as the strongest predictor among the examined demographic and personality factors. This model explains 11.1% of the variance in the dependent variable (R^2^ = 0.111).

The second model includes, in addition to personal accomplishment, the maladaptive coping strategy of self-distraction. This model explains 12.5% of the variability in the dependent variable (R^2^ = 0.125). The third model incorporates personal accomplishment, self-distraction, and the adaptive strategy of religiosity and spirituality (negative regression coefficient in Table 5), explaining 14.0% of the variance in the dependent variable (R^2^ = 0.140).

Other examined demographic and personality characteristics did not contribute to explaining the intensity of moral distress among nurses. The intensity of moral distress experienced by nurses is most strongly predicted by personal accomplishment, self-distraction, and religiosity and spirituality. These represent a combination of selected dimensions of burnout and coping strategies, with religiosity and spirituality potentially acting as a protective factor and an adaptive mechanism for coping with stress.

### Summary of Results

Personal accomplishment, the maladaptive coping strategy of self-distraction, continuous shift operation, and emotional burnout appear to be significant predictors of the frequency of moral distress among nurses. For the intensity of moral distress, significant predictors include personal accomplishment, the maladaptive strategy of self-distraction, and the adaptive strategy of religiosity and spirituality, which operates in the opposite direction and appears to function as an adaptive coping mechanism for dealing with the intensity of moral distress.

## 4. Discussion

This study focused on evaluating selected demographic and personality factors as predictors of the occurrence of moral distress among nurses in clinical practice within the sociocultural context of Slovakia. The findings demonstrated that several of the examined factors emerged as predictors of the occurrence and level of experienced moral distress among nurses. Although their combined effect was relatively modest, they emerged as statistically significant predictors.

Specifically, these included aspects of burnout, particularly the dimensions of personal accomplishment and emotional exhaustion. An interesting finding is the significance of the personal accomplishment dimension, which emerged as the strongest predictor of both the frequency and intensity of moral distress. This result may suggest that nurses who perceive higher demands on their own performance or who more intensely reflect on their professional role may be more sensitive to ethical conflicts in practice. In situations where it is not possible to achieve the ideal level of care, a discrepancy may arise between expectations and reality.

A study in 2017 identified that moral distress significantly and positively correlated with all three dimensions of burnout [9]. Another study reported that moral distress negatively correlated with social support and psychological resilience and positively correlated with emotional exhaustion, depersonalization, and reduced personal accomplishment and that moral distress had a significant impact on burnout syndrome [40]. Similar findings are reported in research conducted in 2025 [41], where moral distress was shown to directly influence burnout. Other authors also confirm that the severity of burnout correlates with higher levels of moral distress [42]. Burnout-related factors associated with higher levels of moral distress were present both before and after the experience of moral distress, suggesting a vicious cycle, as burnout intensifies moral distress and is simultaneously reinforced by it [43]. As moral distress increases, emotional exhaustion and depersonalization also increase, even when accounting for spiritual well-being [44].

Significant correlations have also been found between spiritual well-being and dimensions of burnout, as well as between spiritual well-being and moral distress, which is consistent with our findings. A 2023 study demonstrated an association between the intensity of moral distress and dimensions of emotional exhaustion, depersonalization, and reduced professional accomplishment. Nurses with high emotional exhaustion and depersonalization exhibited a higher intensity of moral distress across both the general scale and six specific factors (recognition, power, and professional identity; safe and competent care; advocacy of values and rights; working conditions; ethical violations; and work teams). Conversely, nurses with low professional accomplishment and burnout showed higher intensities of moral distress only in factors related to recognition, power, and professional identity, which aligns with our findings [16].

Coping strategies also emerged as statistically significant predictors. The coping strategy of self-distraction proved to be a significant predictor, consistent with previous analyses highlighting the negative role of avoidant coping mechanisms. Although such strategies may temporarily reduce psychological tension, they do not contribute to resolving ethical conflicts in the long term, which may lead to the persistence or even escalation of moral distress. Coping styles focused on leaving problems unresolved, problem-focused coping, and lack of formal power have also been identified as predictors of moral distress.

In contrast, the coping strategy of religiosity and spirituality emerged as a negative predictor in the model of moral distress intensity, indicating its potential protective function. This finding supports the interpretation that spirituality may represent an internal coping resource that reduces the emotional impact of ethically challenging situations [45]. In relation to religion, spiritual practices were not perceived as sufficient to manage moral distress among bedside nurses [46]; however, increased spiritual intelligence has been shown to significantly reduce moral distress and compassion fatigue [47]. The role of spirituality is further supported by research conducted in 2025, which indicates that positive environmental and individual spiritual factors significantly influence and reduce nurses’ moral distress, emphasizing the importance of spiritual education and environmental strategies to support a spiritually supportive healthcare environment [48].

Additionally, shift work, particularly continuous operation, emerged as a statistically significant predictor. This represents a work-related characteristic that may be interpreted in the context of increased workload, disrupted circadian rhythms, and a higher likelihood of exposure to stressful and ethically challenging situations during shifts. This finding supports the assumption that organizational and working conditions play a significant role in the development of moral distress. The work environment, working hours, professional engagement, and autonomy have also been identified as predictors of moral distress [49].

Despite identifying several statistically significant predictors, the overall explained variance of the models remains relatively low (11–16%), highlighting the multifactorial nature of moral distress. These findings suggest that other factors likely play a key role.

In our study, other researched sociodemographic and personality factors were not found to be significant predictors. Similarly, other authors did not identify an effect of demographic factors on the level of experienced moral distress among nurses [50]. In contrast to our findings, other studies [51,52] identified age as a significant predictor, associated with reduced burnout, noting that with increasing age, these adverse outcomes tend to decrease. Other research examining moral distress in relation to demographic factors also identified gender as a significant predictor [53], with women more likely to exhibit higher levels of moral sensitivity and consequently lower levels of moral distress.

The investigation of predictors of moral distress requires further research, particularly focusing on work-related factors or their interaction with personality variables. Recent research suggests that triggers of moral distress can be categorized into three levels: individual (capacity to provide care, task planning, and decision-making ability based on experience), social and relational (responsibility and advocacy for patients and their families), and organizational (factors influencing moral well-being such as policies, support, workload, culture, and resources) [54].

Moral distress has significant consequences not only for nurses themselves but also for the quality of care provide. Factors contributing to missed nursing care are also predictors of the intensity and frequency of moral distress. Therefore, the reasons for missed nursing care may increase both the frequency and the level of moral distress [55]. A similar pattern can be observed in patient and family satisfaction, where an inverse relationship exists between the frequency of moral distress and family satisfaction, as well as adherence to patients’ rights [56,57]. In terms of work-related outcomes, a consistent association has been demonstrated among moral distress, burnout, and the intention to leave the workplace or profession, which, in turn reduces staff retention [58,59]. These findings highlight the need for systematic interventions aimed at reducing moral distress, not only from the perspective of protect nurses’ mental health but also to ensure the quality and continuity of healthcare delivery.

Moral distress can be mitigated through organizational support and education, organizational guidelines, unit-specific guidelines, formal forums for discussing ethically challenging situations, ethics-focused education, peer support, watching films, communicating with family and friends, taking walks, maintaining a sense of humor, yoga, meditation, listening to music, adequate nutrition, and sufficient sleep [60,61].

Nursing education in Slovakia is part of the tertiary education system and is harmonized with European Union requirements, particularly Directive 2005/36/EC on the recognition of professional qualifications [62]. The shortage of nurses, combined with retirements and low interest among young people in the profession, has become a significant issue, including in Slovakia. In 2022, Slovakia reported an average of 5.4 nurses per 1000 inhabitants, compared to the EU average of 8.1 per 1000, reflecting an estimated deficit of approximately 15,000 nurses in the Slovak healthcare system [63]. They confirm that physical demands associated with nurse shortages, insufficient work-support tools, time pressure, and challenging working conditions contribute to occupational stress and reduced performance [64].

The limitations of this study include the purposive sampling of the research sample and the use of self-report questionnaires to assess the studied variables. The data were cross-sectional, which restricts the ability to draw causal conclusions. Although regression analyses were conducted to examine associations between the variables, the design does not allow for causal inferences. The relationships identified should be interpreted as correlational rather than causal. Relevant demographic and professional variables were considered; however, the use of stepwise regression limits the ability to ensure full control of potential confounding factors. A limitation of this study is the lower internal consistency observed for several Brief COPE subscales, particularly Active coping, Denial, Behavioral disengagement, Venting, and Self-blame. Although lower reliability is relatively common in two-item Brief COPE subscales, these findings should be interpreted cautiously due to the potential impact of measurement error on the stability of regression estimates. Another limitation is the exploratory use of stepwise regression analysis, which was applied due to the still limited theoretical and empirical evidence concerning moral distress in the Central European post-socialist healthcare context. Therefore, the proposed model should be interpreted cautiously as a preliminary exploratory framework requiring further validation in future research. Among the other limitations were the fact that most participants worked in high emotional intensity settings (e.g., ICU), and the lack of data on social factors such as income and culture, all of which may affect moral distress.

A response rate of 37.45% may represent a limitation regarding the representativeness of the study sample and may increase the risk of self-selection bias. Given the nature of the investigated topic, it cannot be excluded that nurses experiencing higher levels of moral distress were more motivated to participate in the study or, conversely, declined participation due to psychological burden or work-related exhaustion. Despite these limitations, the study provides important insights into factors contributing to the experience of moral distress among nurses and supports the development of effective strategies to prepare nurses for managing moral distress in clinical practice.

### Implications for Clinical and Organizational Practice

The study findings highlight the need for a systematic approach to addressing moral distress at multiple levels of healthcare practice. In clinical practice, it is essential to strengthen nurses’ ability to manage ethically challenging situations, particularly through the development of adaptive coping strategies and the promotion of psychological resilience. The identification of maladaptive strategies, such as distraction, underscores the need for targeted interventions aimed at more effective stress management. At the same time, the results emphasize the importance of burnout prevention, especially in the dimension of personal accomplishment, which was identified as a significant predictor of moral distress.

At the organizational level, the findings have important implications for the management of working conditions, particularly with regard to shift work, which was identified as a significant factor influencing the frequency of moral distress. Optimizing work schedules, reducing workload, and stabilizing teams may help decrease exposure to ethically challenging situations. Another important finding is the insufficient presence of ethics education, highlighting the need for its systematic integration into continuing professional development. The Slovak context extends existing knowledge on moral distress primarily by emphasizing the importance of organizational factors, identifying deficits in ethics education, and confirming the interaction between individual and systemic determinants. The findings also support integrative models of moral distress as a context-dependent and dynamic phenomenon.

## 5. Conclusions

Significant predictors of moral distress among nurses include several aspects related to burnout, coping strategies, and shift work. Personal accomplishment, as a dimension of burnout, appears to be a predictor of both the frequency and intensity of moral distress experienced by nurses, together with the maladaptive coping strategy of self-distraction. Emotional burnout and continuous shift operation are associated with the frequency of moral distress. In terms of intensity, the adaptive strategy of religiosity and spirituality shows a protective effect. These results, in the Slovak context, extend existing knowledge on moral distress primarily by emphasizing the importance of organizational factors, identifying deficits in ethics education, and confirming the interaction between individual and systemic determinants. The findings also support integrative models of moral distress as a context-dependent and dynamic phenomenon.

Identifying predictors of moral distress among nurses is essential for improving their working environment and mental health. Further research in this area is needed to better understand the complexity of moral distress and to develop effective strategies for its mitigation. In this way, ethical practice and the professional development of nurses in healthcare can be strengthened.

## Figures and Tables

**Table 1:** Demographic characteristics of the sample (*n* = 412).

Variable	Category	*n*	%
Gender	Female	391	94.9
	Male	21	5.1
Age	21–29	99	24.0
	30–39	64	15.5
	40–49	152	37.0
	50–69	97	23.5
Marital status	Single	142	34.5
	Married	258	62.6
	Widowed	12	2.9
Religious affiliation	Practicing believer	268	65.0
	Non-practicing believer	99	24.0
	Non-believer	45	11.0
Education	Secondary medical school	103	25.0
	Higher vocational education	48	11.7
	Bachelor’s degree (BSc.)	146	35.4
	Master’s degree (MSc.)	113	27.4
	Doctor of Philosophy (PhD.)	2	0.5
Care unit	Internal medicine department/clinic	68	16.5
	Internal medicine ICU	18	4.4
	Cardiology department/clinic	6	1.5
	Cardiology ICU	7	1.7
	Neurology department/clinic	67	16.2
	Neurology ICU	18	4.4
	Surgical department/clinic	78	18.9
	Surgical ICU	27	6.6
	Department of anesthesiology and intensive care	115	27.9
	Other	8	1.9
Shift work	Single-shift operation	34	8.3
	Two-shift operation (morning and afternoon)	17	4.1
	Three-shift operation (morning, afternoon, night)	38	9.2
	Continuous operation	323	78.4
Worked hours (average over the last 12 months)	Up to 35 h	32	7.8
	36–40 h	277	67.2
	More than 40 h	103	25.0
Training related to ethically and morally challenging situations	Attended training	64	15.5
	Did not attend training	348	84.5

Legend: *n*—absolute frequency; %—relative frequency.

**Table 2:** Overall Evaluation of the Model Predicting the Frequency of Moral Distress Among Nurses.

	R	AdjR^2^	F	*p*
Model 1 (personal accomplishment)	0.356	0.126	59.02	<0.001
Model 2 (personal accomplishment, self-distraction)	0.381	0.145	34.58	<0.001
Model 3 (personal accomplishment, self-distraction, shift work)	0.393	0.155	24.74	<0.001
Model 4 (personal accomplishment, self-distraction, shift work, Emotional burnout)	0.405	0.164	19.87	<0.001

**Table 3:** Regression Coefficients of Predictors in Four Models Predicting the Frequency of Moral Distress Among Nurses.

	B	SE (B)	β	t	*p*
Model 1					
Personal accomplishment	0.03	0.004	0.35	7.68	<0.001
Model 2					
Personal accomplishment	0.03	0.00	0.35	7.71	<0.001
Self-distraction	0.05	0.01	0.13	2.99	0.003
Model 3					
Personal accomplishment	0.03	0.004	0.34	7.42	<0.001
Self-distraction	0.05	0.01	0.13	2.95	0.003
Shift work	0.06	0.03	0.09	2.11	0.03
Model 4					
Personal accomplishment	0.02	0.00	0.20	4.804	<0.001
Self-distraction	0.04	0.01	0.11	2.51	0.01
Shift work	0.07	0.03	0.10	2.33	0.02
Emotional burnout	0.009	0.004	0.12	2.15	0.03

Legend: B—unstandardized regression coefficient; SE (B)—standard error of the unstandardized regression coefficient; β—standardized regression coefficient.

**Table 4:** Overall Evaluation of the Model Predicting the Intensity of Moral Distress Among Nurses.

	R	AdjR^2^	F	*p*
Model 1 (personal accomplishment)	0.333	0.11	50.71	<0.001
Model 2 (personal accomplishment, self-distraction)	0.354	0.12	29.07	<0.001
Model 3 (personal accomplishment, self-distraction, religiosity, and spirituality)	0.374	0.14	21.94	<0.001

**Table 5:** Regression Coefficients of Predictors in Three Models Predicting the Intensity of Moral Distress Among Nurses.

	B	SE (B)	β	t	*p*
Model 1					
Personal accomplishment	0.03	0.005	0.33	7.12	<0.001
Model 2					
Personal accomplishment	0.03	0.005	0.33	7.13	<0.001
Self-distraction	0.05	0.02	0.12	2.59	0.01
Model 3					
Personal accomplishment	0.03	0.005	0.32	6.92	<0.001
Self-distraction	0.06	0.02	0.13	2.91	0.004
Religiosity and spirituality	−0.044	0.01	−0.12	−2.61	0.009

Legend: B—unstandardized regression coefficient; SE (B)—standard error of the unstandardized regression coefficient; β—standardized regression coefficient.

## Data Availability

The original contributions presented in this study are included in the article. Further inquiries can be directed to the corresponding author.
